# A Case of Rheumatoid Arthritis Complicated by Compression of the Popliteal Artery and Tibial Nerve Due to an Organized Hematoma in the Popliteal Fossa: A Case Report and Literature Review

**DOI:** 10.3390/diagnostics15101265

**Published:** 2025-05-16

**Authors:** Gi Beom Kim, Min Cheol Chang, Hyun-Je Kim

**Affiliations:** 1 Department of Orthopedic Surgery, College of Medicine, Yeungnam University, 317-1 Daemyung-dong, Namku, Daegu 42415, Republic of Korea; yumcknee@naver.com; 2 Department of Rehabilitation Medicine, College of Medicine, Yeungnam University, Daegu 42415, Republic of Korea; 3 Division of Rheumatology, Department of Internal Medicine, College of Medicine, Yeungnam University, Daegu 42415, Republic of Korea

**Keywords:** rheumatoid arthritis, hematoma, mass, nerve injury, arterial occlusion

## Abstract

Background/Objectives: Among the various types of masses that can cause compression, hematomas are a relatively common but often overlooked cause. Rheumatoid arthritis (RA) is associated with bleeding problems due to vascular inflammation, platelet dysfunction, impaired production of clotting factors, and medication use. Case Presentation: We report a case of a 76-year-old woman with RA who developed vascular and neurological symptoms in her right lower leg due to compression of the popliteal artery and tibial nerve by an organized hematoma in the popliteal fossa. She experienced swelling, pain, and plantar flexor weakness in the affected leg with no history of trauma. Magnetic resonance imaging revealed a mass measuring 1.2 × 1.0 × 3.0 cm in size in the right popliteal fossa that was in contact with the popliteal artery and tibial nerve on its posterolateral aspect. Electrodiagnostic examination revealed that the right tibial neuropathy developed most probably around the knee level. Surgical excision of the hematoma resulted in almost complete resolution of symptoms, and excisional biopsy disclosed findings of an organized hematoma. We confirmed that the patient’s symptoms were induced by compression of the popliteal artery and tibial nerve due to the organized hematoma in the right popliteal fossa. Conclusions: This case report emphasizes the importance of considering space-occupying lesions, such as organized hematomas, in patients with RA who develop neurological and vascular symptoms.

## 1. Introduction

A mass or tumor in the musculoskeletal system can compress various structures, such as nerves, blood vessels, and soft tissue [1,2,3]. Different symptoms can develop depending on the structure that is compressed. For instance, when a nerve is compressed, neuropathic pain, numbness, tingling, or weakness can develop [1,2]. When a blood vessel is compressed, ischemic pain, swelling, or changes in skin color can occur [3]. When soft tissue is compressed, the patient can experience focal pain or a limitation in the range of motion [4]. When these symptoms are present, it is important that clinicians consider compression by a mass as one of the probable causes.

Among the various types of masses that can cause compression, hematomas are a relatively common but often overlooked cause [5]. Hematomas can develop after trauma, surgery, and inflammation, and, in some cases, they can become organized, resulting in the compression of adjacent structures [6]. Rheumatoid arthritis (RA) can cause bruising and other bleeding problems, including hematomas and purpura [7,8,9]. Patients with RA often experience inflammation in blood vessels, which weakens the vessel walls. Furthermore, platelet dysfunction and impaired production of clotting factors in RA can interfere with proper clotting. The use of NSAIDs, corticosteroids, and DMARDs to manage RA symptoms can also elevate the risk of bleeding.

We report a case of a patient with RA involving the development of neurological and vascular symptoms in a unilateral lower leg due to compression of the popliteal artery and tibial nerve caused by an organized hematoma in the popliteal fossa.

## 2. Case Presentation

This case report was approved by the institutional review board of Yeungnam University Hospital. Written informed consent was obtained from the patient for the publication of this case report and the accompanying images. A 76-year-old woman visited the Department of Rheumatology at our university hospital with swelling, weakness, and persistent pain in her right lower leg. She was subsequently admitted to the hospital for further evaluation and treatment. Her symptoms started approximately 2 months earlier without any traumatic history and had gradually worsened. She reported frequent falls when walking. She experienced pain throughout the entire right lower leg that did not follow the typical dermatomes of the peripheral nerves. The intensity of pain was rated as 3 on the numeric rating scale (NRS, 0: no pain, 10: the worst pain imaginable), and it persisted even at rest without any aggravating factors. Regarding her past medical history, she was diagnosed with RA 9 years earlier, characterized by chronic arthritis in both wrists, positive rheumatoid factor and anti-cyclic citrullinated peptide antibody, and elevated erythrocyte sedimentation rate. She had been taking oral medications, including methotrexate (12.5 mg weekly) and hydroxychloroquine (200 mg daily). Regarding bleeding tendency, the patient was not taking antiplatelet or anticoagulant medications.

Upon admission, the patient’s vital signs were as follows: blood pressure 140/70 mmHg, heart rate 70/min, respiration rate 12/min, and body temperature 36.8 °C. Her blood test results were as follows: white blood cell count 8210/μL, hemoglobin level 12.0 g/dL, platelet count 217,000/μL, C-reactive protein 3.87 mg/dL, erythrocyte sedimentation rate 89 mm/h, rheumatoid factor 142.1 IU/mL, anti-citrullinated peptide antibody 500 U/mL, negative antineutrophil cytoplasmic antibody (both proteinase-3 and myeloperoxidase), and antinuclear antibody titer 1:160. However, all other extractable nuclear antigens were negative, including Smith antibody, phospholipid antibody, cardiolipin antibody, SS-A/SS-B, ribonucleoprotein antibody, centromere antibody, Ho-1 antibody, and Scl-70 antibody (Table 1).

Physical examination revealed mild-to-moderate degree swelling in the entire right lower leg without any significant change in skin color. The strength of the right plantar flexor decreased to a muscle manual test (MMT) grade 3, and no sensory change was detected. The deep tendon reflex of her right Achilles tendon decreased. Ankle clonus and flexor plantar reflexes were not present in both legs.

**Figure 1:**
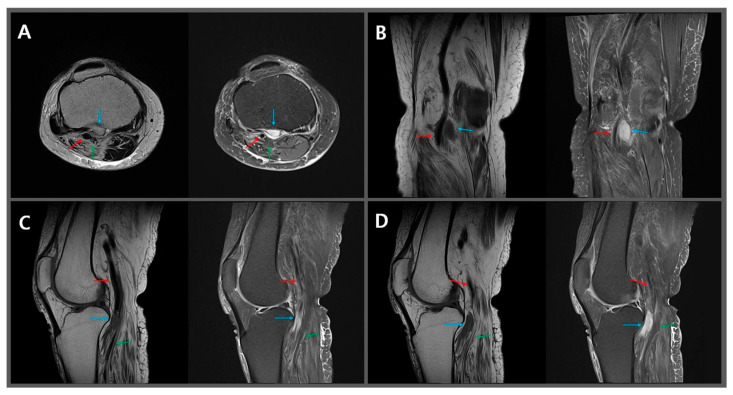
(**A**) Axial T2-weighted (**left**) and proton density fat suppression (**right**), (**B**) coronal T1-weighted (**left**) and proton density fat suppression (**right**), (**C**,**D**) sagittal T1-weighted (**left**) and proton density fat suppression (**right**) MR images reveal that the mass (blue arrow) behind the right knee joint is in contact with the popliteal artery (red arrow) and tibial nerve (green arrow).

A nerve conduction study (NCS) and electromyography (EMG) were conducted. The NCS finding of the right tibial motor nerve with the adductor hallucis muscle revealed a reduction of amplitude (1.5 mV) compared with that in the left tibial nerve (4.2 mV). Moreover, EMG revealed decreased recruitment and a positive sharp wave (1+) in the medial head of the right gastrocnemius. No abnormality was detected in the right biceps long head muscle. Magnetic resonance imaging (MRI) revealed a mass measuring 1.2 × 1.0 × 3.0 cm in size, showing hypointensity on T1-weighted images and hyperintensity on T2-weighted images behind the posterior intercondylar area of the right tibial bone (Figure 1). The mass was encapsulated and in contact with the popliteal artery and tibial nerve on its posterolateral aspect.

As a part of the evaluation for underlying lymphoproliferative diseases, malignancies, vasculopathies, and hidden infection foci, we conducted a positron emission tomography–computed tomography (PET-CT) scan to differentiate between infectious diseases and malignancies. The PET-CT scan revealed uneven mild FDG uptake in several joint areas of both shoulders, elbows, and knees. Moreover, there was no abnormal uptake of 18-F-fluorodeoxyglucose in the scan. A 3D venous CT of the lower extremity performed to evaluate the localized edema in the right lower leg showed no evidence of deep vein thrombosis.

Based on the patient’s symptoms, physical examination findings, and the results of electrodiagnostic and MRI examination, we considered that the mass in the right popliteal fossa would compress the popliteal artery and tibial nerve, which could be the cause of the patient’s vascular (entire lower leg pain with swelling) and neurological (weakness on plantar flexor) symptoms.

**Figure 2:**
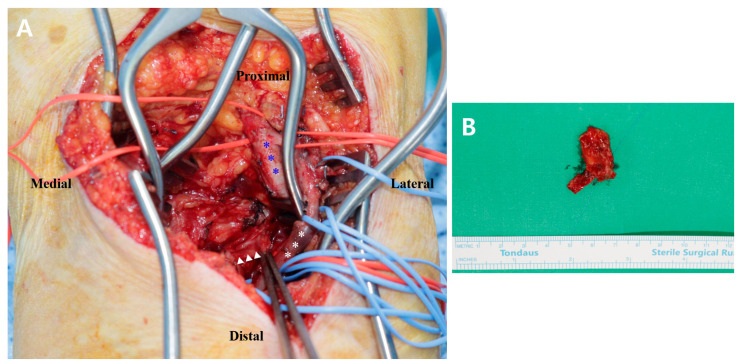
(**A**) Intraoperative photographs depicting the deep posterior approach performed in the right knee. After retraction of both gastrocnemius muscle heads and popliteal artery (blue asterisks) and the vein (white asterisks) using a self-retractor, a mass-like lesion (white arrow heads) with a size of approximately 1.2 × 1.0 × 3.0 cm, adjacent to the popliteal vessels, was exposed and (**B**) excised.

We placed the patient in the prone and slightly flexed knee position under general anesthesia. The iliac crest was padded to prevent pressure sores, and a small bump was placed under the ankle to create a slightly flexed knee position. A pneumatic tourniquet was applied to the upper thigh of the affected leg. The leg was draped in a sterile manner, and the tourniquet was inflated. A “Z”-shaped incision was made, starting along the medial border of the semitendinosus (at approximately 7 cm proximal to the flexion crease) and extending to 8 cm distal to the flexion crease at the medial border of the lateral gastrocnemius muscle. The subcutaneous tissue was retracted, effectively exposing the underlying fascia. The fascia was incised to expose the lateral head of the gastrocnemius. Proximal and distal self-retractors were used to retract both gastrocnemius heads and the neurovascular bundle laterally. Finally, a mass-like lesion with a size of approximately 1.2 × 1.0 × 3.0 cm, adjacent to the neurovascular bundle, was exposed and removed (Figure 2). Active range of motion exercise was started on the first postoperative day. If the acute pain subsided, partial weight-bearing with a crutch was started on the second postoperative day. Complete weight-bearing was allowed at 1 week postoperatively.

Excisional biopsy revealed an organized hematoma accompanied by chronic inflammation, neovascularization, and hemosiderin deposition. The polymerase chain reaction test for mycobacterium and nontuberculous mycobacterium in the tissue showed negative results.

After 1 week of surgery, the patient’s pain and swelling completely resolved, and the plantar flexor strength improved from MMT grade 3 to 4. Following complete healing of the surgical wound, suture removal was carried out. The patient is currently under outpatient follow-up in the department of rehabilitation medicine to facilitate recovery of plantar flexion and continues to receive treatment for RA in the department of rheumatology.

## 3. Discussion

We describe a case in which the patient developed vascular and neurological symptoms caused by compression of the popliteal artery and tibial nerve due to an organized hematoma in the popliteal fossa. The compression of the popliteal artery induced swelling and ischemic pain in the patient’s right lower leg, and the compressed tibial nerve caused weakness in the right plantar flexor.

The organized hematoma formation in our patient may be attributed to RA. RA is characterized by inflammation and hyperplasia of the synovial membrane [10]. In synovial tissues, endothelial activation results in the formation of synovial microvessels, causing synovitis and an increase in the formation of blood vessels [10]. Inflammation in the knee joint could damage the blood vessels in the synovial membrane, resulting in bleeding and hematoma formation. This hematoma can accumulate within the joint or surrounding tissues and can potentially compress the adjacent structures. Especially when the hematoma becomes organized, it hardens, increasing the pressure exerted on the adjacent structures, which can cause more severe symptoms due to the intensified compression on the vessels or nerves near the organized hematoma.

The hematoma observed on MRI revealed hypointensity on T1-weighted images and hyperintensity on T2-weighted images, indicating that the hematoma is chronic [11]. This is consistent with the fact that our patient’s symptoms initiated 2 months before the MRI. Furthermore, although the magnetic resonance images demonstrated that the popliteal artery and tibial nerve are almost in contact with the organized hematoma, it remains unclear whether there is compression. Nevertheless, the swelling on the affected lower leg and the fact that the patient’s pain did not follow the typical dermatomes of the peripheral nerves suggest the occurrence of vascular occlusion. Moreover, the NCS (reduced amplitude of tibial nerve) and EMG (positive sharp wave on the medial head of gastrocnemius, not on the biceps long head) findings indicated that the tibial nerve may be damaged around the knee joint level. These findings suggest that the hematoma in the popliteal fossa mechanically compresses the popliteal artery and tibial nerve. Furthermore, the improvement of the patient’s symptoms after the surgical removal of the hematoma supports the conclusion that the symptoms were caused by the hematoma located behind the knee joint, compressing the popliteal artery and tibial nerve.

RA is a chronic systemic autoimmune disease that mainly affects the joints but can also lead to extra-articular manifestations, impacting various organs and tissues throughout the body [12]. A less recognized but clinically important complication of RA is an increased tendency to bleed, resulting from a complex interaction of pathological mechanisms [13]. Patients with RA often experience spontaneous bleeding episodes, which can appear as intracerebral hemorrhage, hematoma formation in musculoskeletal tissues, and a greater likelihood of bruising [14,15,16]. These bleeding tendencies are caused by several factors, including inflammation, immune system dysregulation, neovascularization, medication side effects, and mechanical stress on the tissues [12,15,17,18,19].

A primary mechanism contributing to the elevated hemorrhage risk in RA patients is RA-associated vasculitis [15]. This condition predominantly affects small- to medium-sized blood vessels, resulting in endothelial cell damage and increased vascular permeability [15]. This inflammatory process weakens the blood vessel structure, making affected patients more prone to spontaneous bleeding episodes. The chronic inflammation linked to RA triggers immune-mediated damage to the endothelium, disrupting vascular integrity [20]. Vasculitis related to RA can manifest as conditions like purpura, skin ulcers, pyoderma gangrenosum, and, in severe cases, major organ involvement with life-threatening complications [21]. Rheumatoid vasculitis involves the deposition of immune complexes in blood vessel walls [22], activating the complement system and initiating an inflammatory cascade that causes endothelial dysfunction and vessel wall fragility [23]. Studies show that RA-associated vasculitis increases the risk of intracranial hemorrhage due to cerebral blood vessel injury [16]. A meta-analysis by Wiseman et al. found that RA patients have a 1.68 times higher risk of hemorrhagic stroke than those without [16], highlighting the significance of vascular inflammation in RA bleeding complications.

Angiogenesis, the formation of new blood vessels, is a key feature of chronic inflammation in RA [24]. In RA, excessive angiogenesis occurs as a response to prolonged inflammation, particularly in synovial tissues [24]. While essential for tissue repair and oxygen supply, these newly formed vessels are often immature and fragile [24], making them prone to rupture and increasing the risk of spontaneous hemorrhage. Overexpression of pro-angiogenic factors like vascular endothelial growth factor and fibroblast growth factor leads to leaky and dysfunctional capillaries lacking proper pericyte support [25]. These capillaries are more susceptible to mechanical stress and minor trauma, increasing the likelihood of hematomas in soft tissues and around joints. The presence of these weak capillaries significantly contributes to hemorrhagic events, especially in periarticular and deep soft tissues [24].

RA involves the accumulation of immune complexes in blood vessels and joints, which damage vessels by activating the complement system and releasing inflammatory cytokines [17,19]. This inflammatory environment causes endothelial dysfunction and increased vascular permeability, making blood vessels more susceptible to bleeding. Key inflammatory cytokines like tumor necrosis factor-alpha, interleukin-1, and interleukin-6 significantly contribute to this process [26]. Elevated levels of these cytokines increase oxidative stress and disrupt the endothelial barrier, leading to endothelial dysfunction and vascular fragility [27]. Additionally, matrix metalloproteinases (MMPs), enzymes from inflammatory cells, degrade the extracellular matrix around blood vessels, further weakening vascular integrity and increasing the risk of spontaneous bleeding [28].

RA treatment, relying on disease-modifying antirheumatic drugs, includes immunosuppressants and various anti-inflammatory agents, which, despite their necessity, can increase hemorrhage risk [19,29]. Nonsteroidal anti-inflammatory drugs and corticosteroids, common for joint pain and inflammation, are particularly implicated [19,29]. Nonsteroidal anti-inflammatory drugs like ibuprofen and naproxen inhibit platelet aggregation by blocking cyclooxygenase, impairing hemostasis, and raising the risk of spontaneous bleeding [30]. Corticosteroids, another RA mainstay, weaken blood vessels by thinning walls and impairing collagen synthesis [31]. Prolonged use is linked to increased skin and mucosal hemorrhages due to compromised vascular integrity [32]. Corticosteroids can also cause osteoporosis and fracture susceptibility, indirectly leading to soft tissue hemorrhage after minor trauma [33]. Many RA patients also take anticoagulants (e.g., warfarin, heparin) or antiplatelet agents (e.g., aspirin, clopidogrel) for cardiovascular conditions like atherosclerosis and atrial fibrillation, further increasing the risk of hematoma and spontaneous bleeding due to the interference with normal clotting mechanisms [19,29].

Chronic RA inflammation leads to progressive joint deformities and periarticular tissue damage [12]. These abnormal joints can mechanically stress adjacent blood vessels, increasing the risk of rupture and hematoma formation. Additionally, RA patients often experience muscle wasting and soft tissue degradation from chronic inflammation and disuse [34], making small blood vessels in weakened tissues easily damaged by minor trauma, resulting in frequent bruising and hematomas. The increased hemorrhage in the musculoskeletal system of RA patients stems from a combination of vascular inflammation, fragile neovascularization, immune dysfunction, medication side effects, and mechanical stress on weakened tissues.

Hematoma, particularly in deep soft tissues and around joints, can significantly compress adjacent structures, leading to nerve compression and vascular obstruction [35,36]. In confined spaces, hematomas can cause mechanical displacement and impair the function of surrounding tissues [35,36]. Large hematomas can directly press on peripheral nerves, causing neuropathic symptoms like pain, numbness, and weakness [37]. Hematomas can also compress blood vessels, leading to impaired circulation and potential ischemic injury in distal tissues [38]. This is especially concerning for RA patients, who already have compromised vascular integrity from chronic inflammation or vasculitis. Prolonged vascular compression can result in hypoxia, necrosis, and increased infection risk. Therefore, RA patients with signs of nerve compression or vascular insufficiency should be evaluated for hematomas, which can be diagnosed using imaging like ultrasound, MRI, and CT scans.

In the present case, it is also necessary to consider rheumatoid vasculitis as a differential diagnosis. Rheumatoid vasculitis is a condition that involves inflammatory lesions in blood vessels with long-standing and severe RA [39]. Rheumatoid vasculitis primarily affects the skin, fingers/toes, and peripheral nerves. It most commonly manifests on the skin as palpable purpura or nonhealing ulcers [40]. A frequent symptom of Rheumatoid vasculitis is vasculitic neuropathy, which occurs due to infarction of peripheral nerves caused by vasculitis of the vasa nervorum [41]. It can involve sensory nerves or both sensory and motor nerve dysfunction and typically presents as mononeuritis multiplex, which is asymmetric and asynchronous and has a tendency to affect distal nerves. The differential symptom points in this case were that although there were abnormalities in the right tibial nerve, the involvement of a single nerve suggests that it is more reasonable to suspect a space-occupying lesion rather than vasculitis.

In the present case, rheumatoid vasculitis should also be considered as a differential diagnosis. Rheumatoid vasculitis is a condition that involves inflammatory lesions in the blood vessels with long-standing and severe RA [39]. Rheumatoid vasculitis primarily affects the skin, fingers/toes, and peripheral nerves. It most commonly manifests on the skin as palpable purpura or nonhealing ulcers [40]. One of the frequent symptoms of rheumatoid vasculitis is vasculitic neuropathy, which occurs due to infarction of the peripheral nerves caused by vasculitis of the vasa nervorum [41]. It can involve sensory nerves or both sensory and motor nerve dysfunction and typically presents as mononeuritis multiplex, which is asymmetric, asynchronous, and has a tendency to affect distal nerves. The differential symptom points in this case were that although there were abnormalities in the right tibial nerve, the involvement of a single nerve suggests that it is more reasonable to suspect a space-occupying lesion rather than vasculitis.

## 4. Conclusions

We report a case of a patient with RA in whom compression of the popliteal artery and tibial nerve by an organized hematoma in the popliteal fossa resulted in symptoms of ischemic pain, swelling, and plantar flexor weakness in the affected lower leg. Surgical removal of the hematoma significantly improved the symptoms, thus confirming the diagnosis. This case emphasizes the importance of considering space-occupying lesions, such as an organized hematoma, as a potential cause of neurological and vascular symptoms, especially in patients with underlying conditions, such as RA.

## Figures and Tables

**Table 1:** Laboratory findings of this case.

	Test Results	Unit	Reference Range
WBC	8.21	/µL	4.0–10
Hb	12.0	g/dL	12–16.5
Platelet	217	/µL	140–440
ESR	89	mm/h	0–20
CRP	3.87	mg/dL	0–0.5
AST	21	U/L	10–35
ALT	15	U/L	10–40
BUN	16.1	mg/dL	8–23
Creatinine	0.45	mg/dL	0.7–1.2
LDH	216	U/L	0–250
Albumin	3.57	g/dL	3.5–5.0
A/G ratio	1.3		1–2.1
IgG	908	mg/dL	700–1600
HbA1c	6.2	%	4.4–6.3
MPO-ANCA	Negative	U/mL	0–5
PR3-ANCA	Negative	U/mL	0–5
ANA	1:160		Negative
RF	142	IU/mL	0–15
ACPA	500	U/mL	0–16
SS-A Ab	Negative (1.0)	U/mL	0–15
SS-B Ab	Negative (2.1)	U/mL	0–15
Scl-70 Ab	Negative	index	Negative
Anti-Jo-1 Ab	Negative	index	Negative
Smith Ab	Negative (0.6)	U/mL	0–15
Anti- dsDNA IgG/IgM	Negative (1.6/4.3)	U/mL	0–20

WBC: white blood cell, Hb: hemoglobin, ESR: erythrocyte sedimentation rate, CRP C-reactive protein, AST: aspartate aminotransferase, ALT: alanine transaminase, BUN: Blood urea nitrogen, LDH: Lactate dehydrogenase, HbA1c: glycated hemoglobin, MPO-ANCA: myeloperoxidase-specific antineutrophil cytoplasmic antibody, PR3-ANCA: proteinase 3-specific antineutrophil cytoplasmic antibody, ANA: antinuclear antibody, RF: rheumatoid factor, ACPA: anti-cyclic citrullinated protein antibody, SS-A: anti-Sjogren’s syndrome-related antigen A autoantibodies, SS-B: anti-Sjogren’s syndrome-related antigen B autoantibodies, Scl-70 Ab: antitopoisomerase-1 antibody, Anti-dsDNA: anti double-stranded deoxyribonucleic acid).

## Data Availability

The datasets generated and/or analyzed during the current study are not publicly available due to privacy, but are available from the corresponding author on reasonable request.
